# Use of Automatic Target Recognition System for the Displacement Measurements in a Small Diameter Tunnel Ahead of the Face of the Motorway Tunnel During Excavation

**DOI:** 10.3390/s8128139

**Published:** 2008-12-10

**Authors:** Jure Klopčič, Tomaž Ambrožič, Aleš Marjetič, Sonja Bogatin, Boštjan Pulko, Janko Logar

**Affiliations:** University of Ljubljana, Faculty of Civil and Geodetic Engineering, Jamova 2, 1000 Ljubljana, Slovenia

**Keywords:** Exploratory tunnel, 3D displacement measurements, automatic target recognition (ATR)

## Abstract

During construction of the Šentvid tunnel a unique opportunity arose to measure the 3D displacements ahead of the motorway tunnel excavation face, since the exploratory tunnel was already constructed in the axis of the main tunnel. According to reviewed literature such measurements had not been performed yet and several problems regarding equipment and complete scheme of the experiment needed to be overcome. The paper gives a brief description of the Šentvid tunnel project, presents significant factors that affected the choice of the geodetic equipment and describes the scheme of the experiment. A special attention is focused on the problems relating to the operation of the instrument in demanding environmental conditions (water, dust).

## 1. Introduction

Construction of a tunnel or any other underground structure depends strongly on an observational method, especially if the structure is constructed under difficult geological conditions [1]. Several methods are available nowadays to observe the underground structure response due to the stress redistribution around an opening, caused by its excavation [2]. The deformations of the underground structure can be monitored using geodetic and/or geotechnical methods. Geodetic methods like terrestrial laser scanning and 3D total station measurements are used for monitoring the convergence of the circumference of the underground structure in absolute coordinates, while the geotechnical methods enable recording of the relative displacements in the surrounding rock [3]. Most common geotechnical measurements are performed with geotechnical instruments such as single- and multipoint extensometers, sliding micrometers, inclinometers, *etc.* [2]

Terrestrial laser scanning allows monitoring of the entire contour of an opening, but lacks accuracy. Due to uneven surface of the lining (usually shotcrete) the accuracy is in the range of a few centimeters and usually does not satisfy the accuracy requirements. However, the advantage of recording the absolute position of a very large number of points sometimes outweighs relatively low accuracy.

In comparison to the laser scanning the 3D geodetic measurements of the optical reflector targets using total station with integrated distance measurement give information on the underground structure response in selected points only. General accuracy of this method is rather higher if compared to the laser scanning and is in the range of a few millimeters [2], depending on the accuracy of the applied geodetic instrument, the type of the reflectors and the distance to the reflectors [4] and the conditions on the site (presence of dust in the area, the size of opening, the length of tunnel). The number of the targets that are mounted on the primary lining in each of the measuring sections and the distances between consecutive measuring sections depend on the geological conditions and the size of an opening; typical distance is 1-2 tunnel diameters [5]. Due to high accuracy the 3D displacement measurements with total station geodetic instruments have become an everyday practice at construction sites around the world.

For complete knowledge on the response of the underground structure one has to be aware also of the magnitude of the displacements that occur ahead of the excavation face (pre-displacements). The differences between the measured displacements in the tunnel compared to the measurements of the surface settlements above the tunnel with low overburden clearly indicate a large portion of pre-displacements [6]. Experimental measurements [7] indicate that more than 30% of displacements occur ahead of the excavation face. These displacements cannot be measured with conventional geodetic equipment. Several geotechnical methods can be/were used to measure the effect of the face approach to the observed cross section (horizontal inclinometer [8], multi-point extensometer, chain deflectometers e.g. multi-element sliding curvometer). As already mentioned, all these methods provide only relative displacements of the surrounding rock.

The 3D displacement measurements ahead of the tunnel face can only be obtained by geodetic monitoring, when a small diameter tunnel exists within the alignment of the future tunnel with considerably larger cross section. Such opportunity arose during the construction of the Šentvid tunnel and a comprehensive monitoring scheme was established. A brief presentation of the Šentvid tunnel project and the details on the execution of the experiment and equipment are given in the sequel.

## 2. The Šentvid tunnel

### 2.1. Description of the project

**Figure 1.**
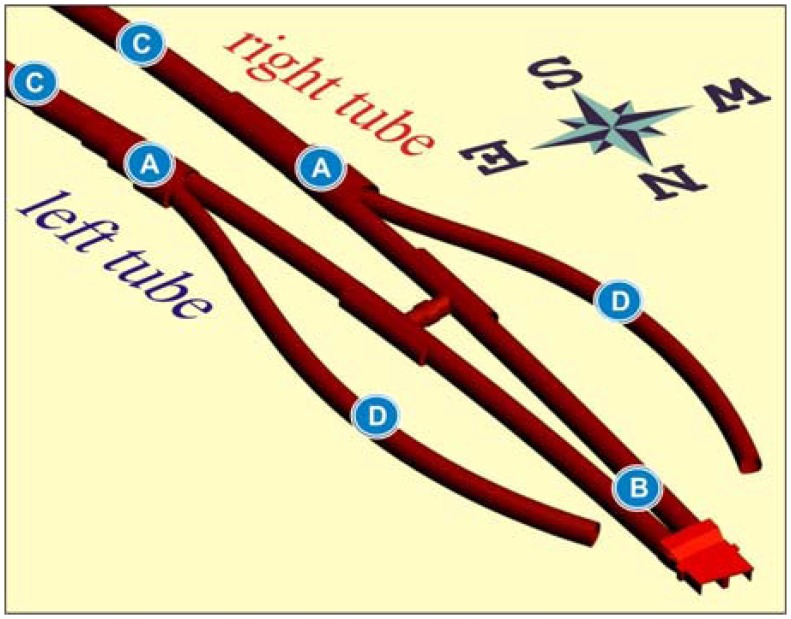
Scheme of the Šentvid tunnel [9].

The Šentvid tunnel system links the Slovenian A2 Karavanke-Ljubljana motorway to the Ljubljana ring motorway. A 1,060 m long motorway tunnel is designed as a double tube tunnel with two large merging caverns with a maximum excavation cross section of approximately 330 square meters and a length of 60 m (label A in Figure 1). The Šentvid tunnel consists of twin two-lane tunnels (cross section of 90 square meters) from northern portal up to the merging caverns (label B) and twin three-lane tunnels (cross section of 135 square meters) from southern portal to the merging caverns (label C). Two ramp tunnels (label D) connect one of the main roads of Ljubljana to the main motorway tunnel. All underground structures were constructed with shotcrete method. Maximum overburden reaches 115 m.

The Šentvid tunnel alignment passes through densely foliated clastic sedimentary rocks of carboniferous age, mainly sandstones, siltstones and clayey slates. The region has undergone intense tectonic deformations, presumably during several deformation phases. Due to intensive tectonics the rock is folded, the fault zones are up to several meters thick and consist mainly of gouge clay. The rock mass itself is very heterogeneous and anisotropic. The tunneling conditions for the Šentvid tunnel system were estimated in the range from fair to very poor [10].

### 2.2. Exploratory tunnel

To determine the most favorable position of the caverns in terms of geological and geotechnical criteria, the exploration gallery in the axis of the main tunnel was constructed in the final stage of the design. According to the geological model assessed with geological mapping and core drilling the beginning of the left cavern was foreseen 369 m (reserve position 453 m) and the beginning of the right cavern 480 m from the northern portal [11]. The alignment of the exploratory tunnel was not precisely defined and depended on the actual geological and geotechnical conditions of the rock mass [9].

**Figure 2.**
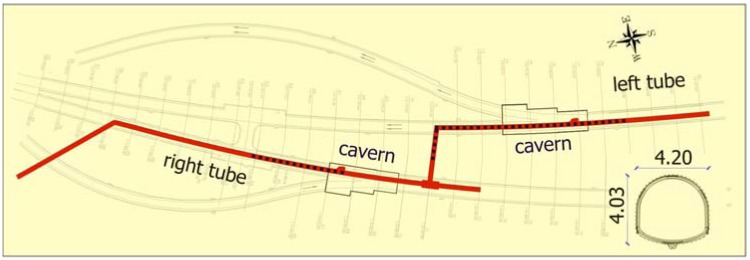
Ground plan and cross section of the Šentvid exploratory tunnel.

The construction of the exploratory tunnel started in April 2004 at the northern portal with the 90 m long access gallery towards the axis of the right tube, as seen in Figure 2, and followed the alignment for approximately 300 m. At a distance of 239 m from the northern portal the exploratory tunnel excavation face entered a large block of sandstone with fair geological and geotechnical characteristics for the construction of the right merging cavern. Afterwards excavation continued through 40 m long cross passage to the left tube. The excavation followed the left tube axis for approximately 150 m to reach the reserve position for the left merging cavern. Based on the measured displacements, mapped lithological units, the degree of fracturing and the degree of tectonisation the decision for the position of the left merging cavern at a distance of 369 m from northern portal was taken. A total length of the constructed exploration gallery was 655 m (red line in Figure 2) and was completed in February 2005. The excavation of the main motorway tunnel started in December 2004 in the left tube and in March 2005 in the right tube. The Šentvid tunnel was given over to the traffic in July 2008.

Regular cross section of the exploratory tunnel (13 m^2^ as seen in Figure 2) depended on the size of the tunneling equipment. The axis of the tunnel raises from the northern portal towards the southern.

The exploratory tunnel allowed the establishment of a reliable geological model and enabled the in-situ geotechnical testing (core drilling, geophysical surveys, extensometers). The geodetic measurements of the 3D displacements during the exploratory tunnel construction improved the knowledge of the rock mass behavior and its response to the tunnel excavation. Further on, the measurements of the 3D displacements in the exploratory tunnel during the construction of the main motorway tunnel were performed.

## 3. Scheme of the experiment and equipment

The main goal of the experiment was to observe the rock mass response ahead of the tunnel face due to the tunnel excavation (displacement range, extension of the influence zone, response when approaching a fault zone, effect of installing rock bolts as a stabilization measure of the face, etc.). Since according to the reviewed literature 3D displacement measurements ahead of the tunnel excavation face had not been performed yet, several problems regarding equipment and complete scheme of the experiment needed to be overcome.

Due to risky environment (possibility of overbreaks at the area of the top heading excavation face and consequently possibility of burying the experiment staff inside the exploratory tunnel) the experiment had to be planned carefully with special attention focused on safety. A requirement to minimize the number of entries of the personnel into the exploratory tunnel on one hand and the request of high precision 3D measurements of large number of points on the other hand influenced the decision regarding the type of the geodetic equipment.

A total station TCRP 1201R300 produced by Leica Geosystems Inc. fulfilled the abovementioned criteria with its Automatic Target Recognition (ATR) sensor that allows automatic angle and distance measurements of prisms. Some successfully accomplished projects using automated monitoring systems have already been reported, for example monitoring of the structures above the King's Cross station during the construction of the tunnel connections [12]. The accuracy of angle measurements of the total station TCRP 1201R300 is ±1” according to the technical data of the instrument, while the accuracy of measuring distances to standard optical prism is ±(2 mm + 2 ppm) and positioniong accuracy of the ATR is below ±2 mm [13]. When performing initial measurements of the points, the prism is sighted with the optical sight. After initiating a distance measurement, the instrument sights the prism centre automatically and both angles and the distance are measured to the centre of the prism. When the initial measurements of all planned points are completed, the program is started with the determination of the regular time intervals of the measurements. The monitoring data are stored to an internal memory card. Power supply was in our case provided with an internal battery as well as with an external battery to extend the operating time of the instrument to more than 24 hours.

**Figure 3.**
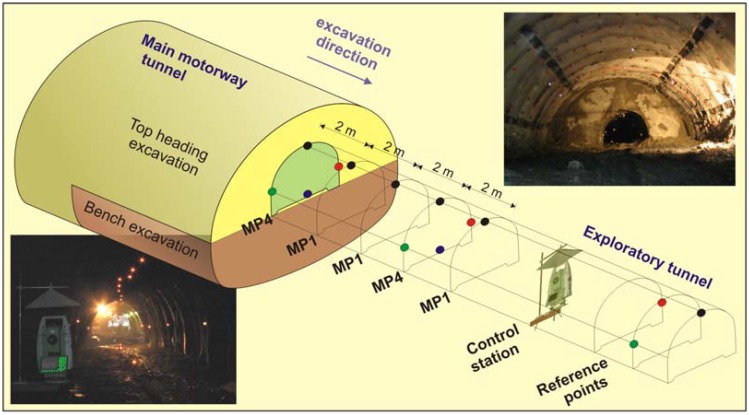
Scheme of the experiment.

To assure high precision measurements, standard optical reflectors had to be mounted on the primary lining of the exploration gallery. The following pattern was selected: optical reflectors were installed every two meters in the crown and every six meters on both side walls and on the ground (Figure 3). A measuring section with single target in the crown was marked with MP1 and the other with 4 targets was marked with MP4. In sections of special interest, such as transition to a fault zone 3 measuring sections with 8 targets (MP8) were installed (3 on the ground and 5 on the primary lining) to obtain more detailed response of the exploratory tunnel. Also of special interest was the behavior of the left wall of the cross passage, when the left tube was excavated in the vicinity. Measuring sections with a crown target and additional target on a left side wall were marked with MP2.

Optical reflectors were mounted on the rebars with diameter of 16 mm that were previously fixed in the primary lining. Since no concrete invert was constructed in the exploratory tunnel, the rebars for the bottom targets were installed directly into the ground and secured with small amount of concrete.

**Figure 4.**
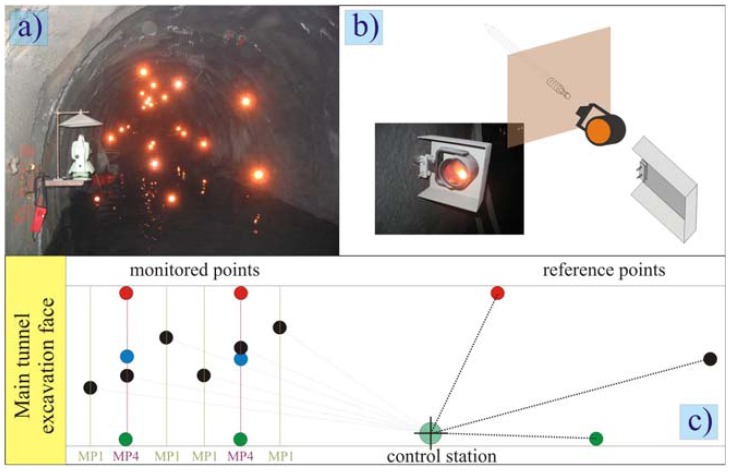
(a) Photo of the disposition of the reflectors in the exploratory tunnel. (b) Detail of the reflector and its protection. (c) Plan view of the monitoring scheme.

A distinction of horizontal and/or vertical angles between the targets in the crown would be insufficient for the use of the ATR due to small distance from each other (only 2 m) and small diameter of the tunnel if the targets were installed in a straight line exactly in the crown. The position of the “crown” point is therefore somewhat changing along the axis (Figure 4a and Figure 4c), depending on the position of the instrument and levelness of the tunnel circumference.

To cover the expected influence zone, due to the excavation of the main motorway tunnel the monitored area was according to literature ([8, 14-15]) primarily determined at 2 diameters of the double lane motorway tunnel ahead of the top heading face (in our case 20 m with 10 measuring sections and approximately 20 targets). The monitored area was further on re-defined regarding the measured displacements of the targets. Zero readings of the positions of the targets were normally taken when the points were ahead of the influence zone in order to record the entire displacement history of each individual point.

To minimize the risk of instrument damages due to the shotcrete application during the primary lining installation in the main motorway tunnel, the distance of the geodetic instrument to the top heading face was never less than 18 m. The instrument was installed on a steel cantilever beam (Figure 4a) on the left or right side wall of the exploratory tunnel, depending on the visibility of the previously mounted targets and the curvature of the tunnel axis. A steel cover was placed above the instrument (see Figure 3, photo in the bottom left) for protection against falling pieces of the primary lining in case of larger cracks. The steel cover was placed also on the optical reflectors that were close to the face to prevent any accidental movements or breakage (Figure 4b).

One of the important issues when planning the experiment was the position of the reference points and the determination of the position of the control station from the known positions of the reference points. According to the previously acquired tunnel surveying experience, three reference points were placed always in the same order: one target on the same side wall as the instrument and one target on the opposite side wall; the distance from these two points to the instrument should be nearly equal and the horizontal angle between the two points as large as possible. The third reference point was installed in the crown deeper in the exploratory tunnel (at a larger distance from the instrument). Initial positions of the first set of reference points were determined from the geodetic network in the main motorway tunnel. Further on, a new set of reference points was determined from the known positions of points monitored at that time. The reference points needed to be installed in a part of the exploration gallery that was not yet influenced by excavation works of the main motorway tunnel top heading excavation face.

The position of the control station was determined at the beginning of each set of angles due to the possible movements of the instrument's plinth (the analysis of some control stations in the left tube of the exploration gallery revealed quite large displacements). Another possibility would be to determine the position of the control station only at the beginning of the measurements at each replacement of the batteries or downloading of the monitoring data. This would actually extend the durability of the batteries, but if the instrument was in the influence zone of the excavation works, the obtained measurements of the monitored points would include a systematic error.

According to the original plan the measurements should be performed in 30 minute intervals, but the analysis of the measured displacements indicated that so frequent measurements were unnecessary and the interval was extended to 60 minutes.

All measurements of the monitored points and of the reference points were performed in both faces. Due to safety reasons the reference points were recorded first, so the instrument then stopped in the second face, pointing to the reference points and the measuring lenses faced away from the approaching tunnel face.

The built-in software of the instrument skips the target, if it is removed. If the instrument can not measure its position due to dewy surface of the target or due to dusty environment, the instrument stops during measuring and the intervention of the operator is needed. To enable measurements in aggravating environmental circumstances or measurements of the prisms with not perfectly clear surfaces, the instrument features a low visibility mode.

## 4. Execution of the measurements

### 4.1. Environmental conditions and safety issues

During the first entry in the exploration gallery the air pollution was checked for methane, carbon dioxide and carbon monoxide, since the exploratory tunnel had no longer been ventilated after its completion. Surprisingly the air in the exploratory tunnel was fresh, even in the most distant parts. On the basis of later observations of the air ventilation we assume that the temperature differences between the ground (cold water) and the crown of the gallery ran the self ventilation and maintained the air fresh.

An important issue was also a communication with the Contractor. Due to safety reasons the entrance to the exploration gallery was allowed once a day and only when the excavation step was completed, the primary lining installed and the face stability ensured. Usually we entered the exploratory tunnel during drilling for the rock bolts or during shifts. The Contractor's staff was also requested to remove the optical reflectors and their protection prior to the excavation of the round length with the measuring section.

### 4.2. Measurements in the right tube of the exploratory tunnel

At the time of planning the experiment the right tube was excavated in the section with the exploratory tunnel ahead of the face, while the left was not. Consequently, the targets and the instrument were installed in the right tube and the measurements started on September 6^th^, 2005. In the first two weeks only one measurement a day was performed to get accustomed to the instrument and to set the exact procedures. Continuous monitoring started on September 22^nd^.

From measuring section P1 to P9 (chainage km1.2+54 to km 1.2+73) the main motorway tunnel was constructed in faulted rock mass and large displacements were measured ahead of the face (maximum vertical displacement 9.3 cm and longitudinal displacement 13.3 cm in the excavation direction).

When the fault zone was passed and the top heading excavation face approached the merging cavern and entered the block of sandstone with very good geological and geotechnical characteristics, the measured displacements decreased significantly to less than 1 cm and remained in the range of the measuring accuracy during further excavation in the cavern. Therefore, the continuous monitoring in the right tube ahead of the excavation face was stopped on October 27^th^ and the instrument was moved into the left tube that entered the section with the exploratory tunnel ahead of the top heading face.

**Figure 5.**
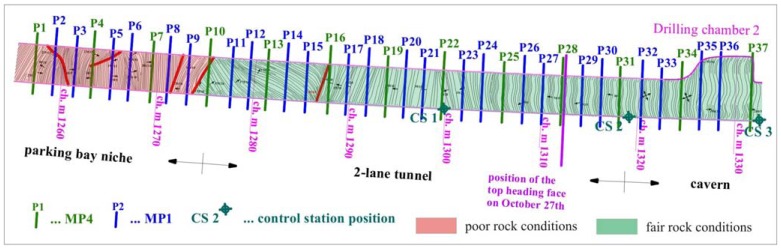
Disposition of the measuring cross sections and control station positions in the right tube of the exploratory tunnel with lined geological layout.

The length of the monitored section in the right tube amounted to 75 m (from chainage km 1.2+57 to km 1.3+32 – the section is plotted with blue dotted line in Figure 2) and included 37 measuring sections (Figure 5). The first 28 measuring sections out of 37 were monitored on complete deformation path (from the time the measuring section got into the influence zone ahead of the face to the excavation of the round length with the measuring section).

**Figure 6.**
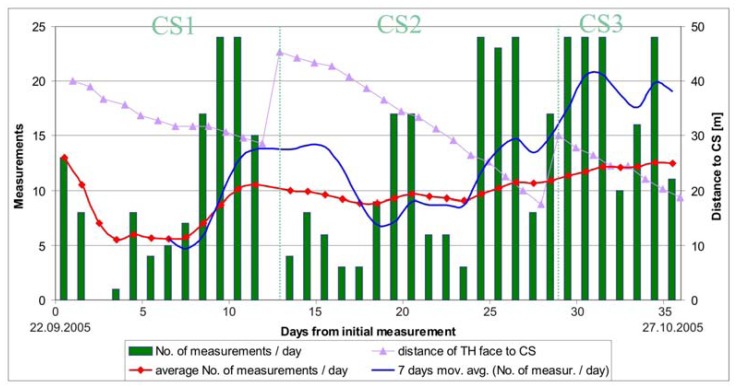
Number of daily performed measurements in the right tube of the exploratory tunnel with plotted average measurement rate and distance of the control station to the top heading excavation face of the main motorway tunnel.

Due to fair tunneling conditions and only minor quantities of water no major problems were encountered during continuous monitoring in the right tube. In 35 days of continuous monitoring 437 sets of measurements were performed or 12.5 measurements per day on average. The number of daily performed measurements through the monitoring period is given in Figure 6.

To avoid accidental damage of the instrument, the first total station position was set at a large distance from the first measuring section (42 meters). With further experience this distance was decreased, since due to the dust caused by the excavation works, the instrument was not able to define the position of the monitored points and thus stopped. Since the entrance to the exploratory tunnel was due to safety and logistic reasons limited to once a day, only some sets of measurements were performed a day if the instrument stopped. The instrument was put in operation again on the next day. The dependence of the number of executed measurements on the distance of the instrument from the face can be observed in Figure 6, which clearly shows that the number of the measurements performed per day was in general increasing with decreasing distance from the control station to the face.

### 4.3. Measurements in the cross passage of the exploratory tunnel

As the excavation of the left tube of the motorway tunnel approached the intersection of the cross passage and the beginning of the left tube of the exploratory tunnel, large cracks developed in the primary lining of the exploratory tunnel. Some individual measurements were executed in this area during the continuous monitoring in the right tube.

To observe a response of the rock mass to excavation also perpendicular to the tunnel axis, 3D displacement measurements were performed in the cross passage. The measurements started on October 7^th^, when the distance of the intersection to the excavation face of the left tube was 11 m. According to the mapped deformation of the primary lining it is obvious that some deformation was missed. The measured displacements were somewhat smaller than in the right tube (maximum vertical displacement 2.7 cm, horizontal 1.3 cm and longitudinal 1.8 cm) due to the geological structure. The cross passage is situated in a block of firm, horizontally foliated sandstone and siltstone.

**Figure 7.**
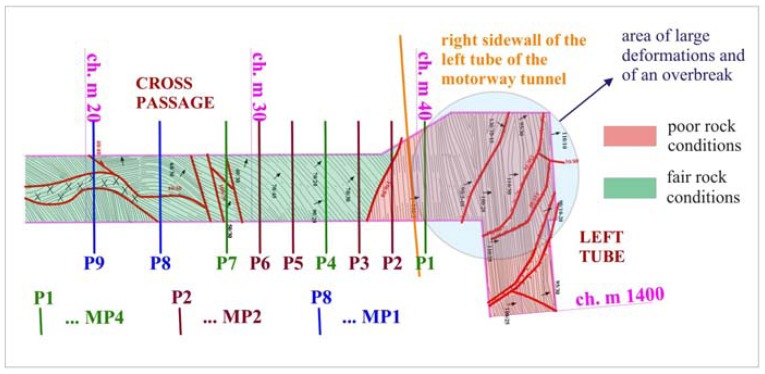
Disposition of the measuring cross sections and control station positions in cross passage of the exploratory tunnel with lined geological layout.

9 measuring cross sections were installed in 20 meter long section in the cross passage, as shown in Figure 7. To minimize the number of measurements on one hand and to cover the expected influence zone on the other, the distance between measuring sections P7 and P8 was 4 meters, while between individual measuring section from P1 to P7 it was 2 meters. The control station was placed in the intersection of the right tube and the cross passage and its position was determined on the basis of the reference points in the right tube of the exploratory tunnel. The measurements were performed once a day till October 20^th^, when the face was 8.6 m ahead of the cross passage. The contractor then reprofiled the entrance to the cross passage. This was an additional contribution to rock mass deformation, as the effects of reprofiling and further excavation of the left tunnel tube acted simultaneously. The original plan to use the cross passage for the observation of the displacements in the perpendicular direction to the advancing tunnel excavation was therefore no longer possible. Thus, the monitoring was stopped.

### 4.4. Measurements in the left tube of the exploratory tunnel

Simultaneously with the measurements in cross passage the monitoring of measuring sections P1 to P13 started also in the left tube of the exploratory tunnel. But only two sets of measurements were executed (October 7^th^ and 10^th^) because of increased influence of the approaching excavation face of the main motorway tunnel and consequently large cracks in primary lining of the exploration gallery. On October 13^th^ an overbreak of approximately 50 m^3^ occurred in this area and the entrance to the left tube of the exploration gallery was temporarily closed. Till October 25^th^ no entry to the exploratory tunnel in the left tube was allowed. Continuous monitoring thus started on October 27^th^, 2005.

Expected as well encountered geological conditions in the left tube were considerably worse than in the right tube. Consequently, the monitored deformations were by a magnitude larger (maximum vertical displacement 26.5 cm, horizontal 16.3 cm and longitudinal 29.3 cm against the excavation direction).

**Figure 8.**
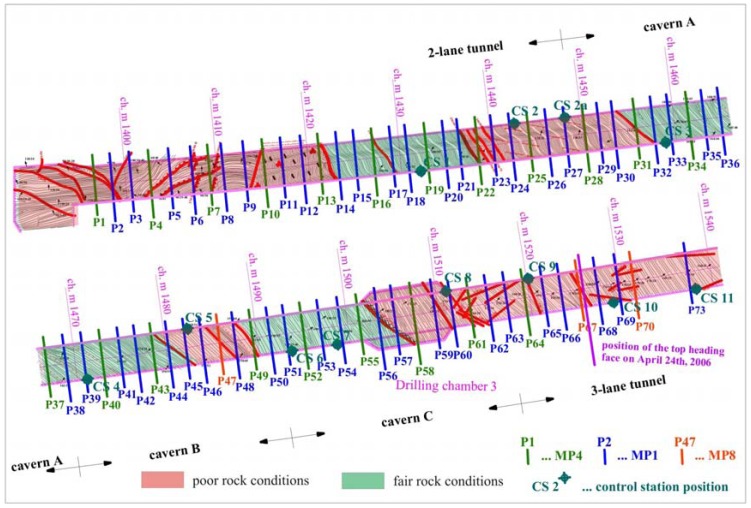
Disposition of the measuring cross sections and control station positions in the left tube of the exploratory tunnel with lined geological layout.

**Figure 9.**
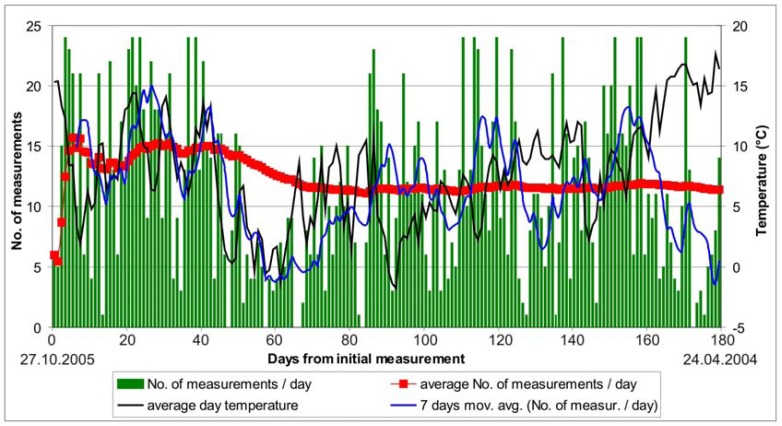
Number of daily performed measurements in the left tube of the exploratory tunnel with plotted average measurement rate and average day temperature in Ljubljana.

A 147 meter long section from chainage km 1.3+97 to km 1.5+44 was equipped with 72 measuring sections, as seen in Figure 8. A complete deformation path of measuring sections from P1 to P66 was obtained. The monitoring of the 3D positions of the targets in the left tube of the exploratory tunnel and generally ahead of the excavation face in the Šentvid tunnel finished on April 24^th^ 2006 due to the worsening of the geological conditions that caused complete deterioration of the primary lining in the exploratory tunnel. 2,040 sets of measurements were performed in 179 days of continuous monitoring in the left tube of the exploration gallery ahead of the excavation face or 11.4 measurements per day on average (the number of performed measurements per day during complete period is plotted in Figure 9).

Unlike the right tube, where dust turned out to be the main problem of the monitoring and only minor quantities of water flowed out of the exploration gallery, in the left tube water and humidity were a major issue. The monitoring in the right tube took place in early autumn with pleasant weather conditions and air that was pumped in the tunnel through the ventilation system was warm and dry. As the outside temperatures decreased in the beginning of December 2005 (black line in Figure 9), the humidity in the tunnel increased and condensed on the optical reflectors, which became wet and covered with droplets of water. The laser beam of the instrument was able to detect a pre-recorded target, but could not measure its position and stopped while measuring. The number of executed measurements per day was drastically reduced, as plotted in Figure 9.

The conditions in the tunnel even impaired from December 20^th^ to January 4^th^, when the construction works were stopped and the construction machinery was not heating the air in the tunnel while running. The optical reflectors were wiped off at every entrance into the exploration gallery, but indispensably became wet again in a few hours. Several liquids and/or procedures to prevent dewy surface of the targets were unsuccessfully applied. The only procedure that worked with limited effect was watering of the prisms and thus making a water film on the surface. The droplets of water arose later and after January 5^th^ a few more sets of measurements were performed daily, as seen in Figure 9.

**Figure 10.**
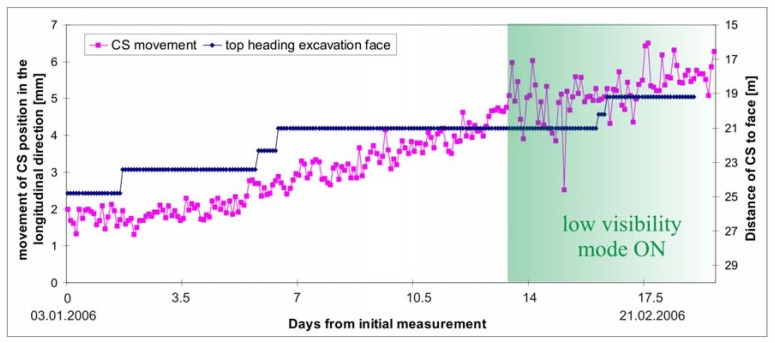
Movement of the position of the control station CS6 in longitudinal direction with plotted distance from control station to the top heading excavation face.

To increase the number of daily performed measurements the low visibility mode was implemented on January 18^th^, but was switched off 6 days later due to rather lower accuracy of the acquired position of monitoring and reference points. Larger scatter up to 3 mm at the determination of the control station position from January 18^th^ on can be seen in Figure 10. From the scatter of measurements in Figure 10 before January 18^th^ the inaccuracy in determining the control station position without low visibility function can be estimated in the range of 1 mm (more precise calculation is given in [16]).

Another problem was related to water damming at the entrance to the exploratory tunnel. The estimated volume of water flowing out of the exploratory tunnel was about 2 L/s (the majority flowed out from the drilling holes in drilling chamber 3 – Figure 8). Due to unfavorable tunneling conditions the excavation works of the top heading face were divided in several smaller steps and usually required several hours to complete. In the time the water was dammed in the exploratory tunnel, it flooded the bottom targets and the instrument stopped if more than half but not the whole of the prism was submerged. The entry to the exploratory tunnel was impossible until the excavated material was removed and the water was drained out.

As already mentioned, the tunneling conditions in the left tube of the Šentvid tunnel were considerably more demanding and the displacements larger than in the right tube. The measurements of the monitoring points also indicated more extensive influence zone ahead of the excavation face and the instrument was situated inside this zone (usually at distance 20-30 m from the excavation face). The displacement of the instrument on the cantilever beam was primarily noticed on the circular level of the instrument and further analysis confirmed movement of the control station as seen in Figure 10. In this case the decision to determine the position of the control station in each set of angles proved to be appropriate. When the influence zone extended to up to 45 m [17], the reference points were also exposed to movements. A correct procedure in this case would be to move the instrument further into the exploration gallery, but due to the levelness and small diameter of the constructed exploratory tunnel this would cause problems with Automatic Target Recognition.

**Figure 11.**
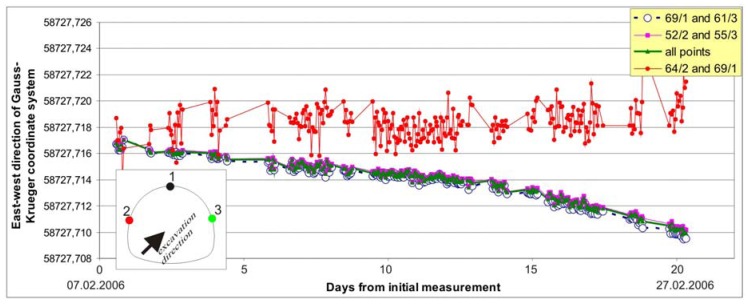
Movement of the position of the control station CS8 in east-west direction of Gauss-Krueger coordinate system as calculated from different combinations of the reference points.

On the basis of the performed measurements we concluded that due to the geological structure the reference point on left side wall would move prior to the point on the opposite wall and the analysis of the control station position confirmed it. In Figure 11 the position of CS8 in east-west direction of Gauss-Krueger coordinate system as calculated from different combinations of the reference points is plotted with time, since known positions of two targets are required for the calculation of the position of the third point. The calculated position with combination crown point – right sidewall point and left sidewall point – right sidewall point is nearly identical, while combination crown point – left side wall point secedes. In this case only crown point and right side wall point were employed in the calculation of the control station position.

At the end of continuous monitoring in the exploratory tunnel ahead of the excavation face of the left tube the position of the latter reference points was measured again from the geodetic network in the main motorway tunnel. After 6 months during which time 147 m of the exploration gallery were monitored and the control station position was changed twelve times, the accumulated difference between the position of the reference points, measured from the main motorway tunnel and from local geodetic network in exploratory tunnel, amounted to 8.5 cm towards west and 3.5 cm towards north. The accumulated height difference was 2 cm.

## 5. Results

**Figure 12.**
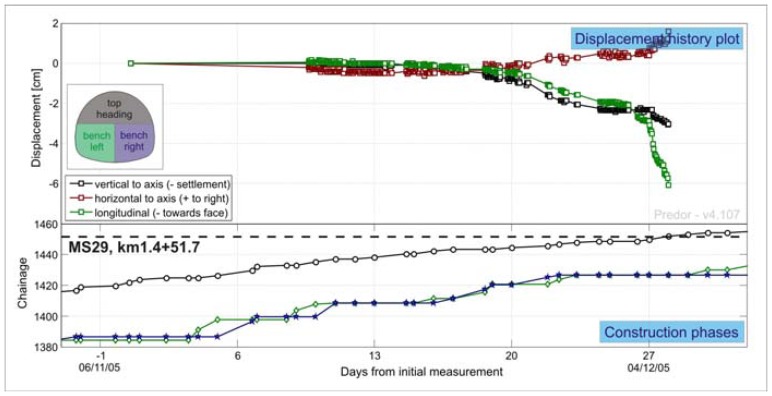
Spatial displacement history plot of the crown point in measuring cross section MS43 at chainage km 1.4+78.6 in the left tube of the exploratory tunnel.

A simple 3D displacement history plot of a target ahead of the approaching main tunnel excavation face (Figure 12) is the basic outcome of the presented experiment. The obtained curve in vertical and longitudinal directions is of parabolic shape as presumed by Barlow [15] on the basis of numerical calculations. The displacement curve in the horizontal direction is somewhat exceptional, but can be explained with the foliation of the surrounding rock mass [17].

The comparison of the displacements in the exploratory tunnel and in the main tunnel revealed that in the monitored section of the left tube of the Šentvid tunnel the measured displacements ahead of the face amounted to between 15% and 45% of the total measured displacements in the same cross section and depended on the stiffness of the rock mass [17].

**Figure 13.**
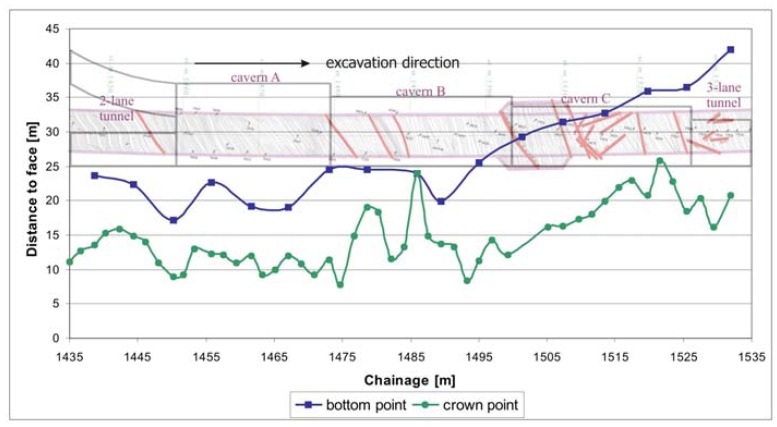
Extension of the influence area ahead of the face for crown and bottom point.

One of the main goals was also to determine the influence zone extension ahead of the excavation face. The area of large pre-displacements extends about a half of tunnel diameter ahead of the face, while the maximum distance of recognizable displacements to the face reached up to 45 m (Figure 13). To eliminate the measurement accuracy a threshold of 3 mm was selected as recognizable displacement.

## 6. Conclusions

The exploratory tunnel that was constructed in the axis of the future motorway tunnel offered a unique opportunity to measure 3D displacements ahead of the motorway tunnel excavation face. Geodetic measurements were performed with the total station TCRP 1201R300 (Leica Geosystems Inc.) equipped with Automatic Target Recognition system. This system allowed to minimize the number of somewhat risky entries into the exploratory tunnel and still perform several, ideally 24 measurements per day. Despite extremely demanding working environment (dust, humidity) the system performed well during 7 months of operation and, in our opinion, presented an optimum choice for the task. The accuracy of the determination of the control station position was about 1 mm [16], despite the fact that the total station had to be placed close to the monitored points, generally within the influence area of the tunnel excavation in order to get the highest number of executed measurements. The number of daily performed measurements ranged from 1 to 24, with an average value of 11.7, and it depended on dust, humidity, temperature, occasional accumulations of water on the floor of the gallery and on the distances from the total station to individual reflectors. The internal and external batteries provided reliable and sufficient source of energy for more than 24 hours when measuring 25 reflectors in both faces once per hour.

The weakest point of the system was in our case the Automatic Target Recognition system. It worked well if the reflectors were clean and dry and even if certain reflector (or group of them) were not visible or removed. However, the system stopped and needed an operator intervention if the target was found but no accurate measurement could be obtained due to droplets of water, dust, and in some cases when the reflector was partially submerged. Both batteries were completely exhausted in these cases.

This unique full scale experiment provided valuable data on rock mass behavior ahead of the tunnel face in weak anisotropic rock mass conditions, even though it was not able to provide measurements in the areas with the highest deformations, since the entry for the research staff was not allowed in such sectors for safety reasons.

## Figures and Tables

**Table 1.** Summary of performed 3D displacement measurements in the exploratory tunnel

	**chainage**	**Section length [m]**	**No. of meas. sections**	**Monitoring days**	**Sets of meas.**	**Avg. No of sets of meas. / day**	**Maximum displacement [cm]**		

							**Vert.**	**Horiz.**	**Long.**
Right tube	Km 1.2+57 – km 1.3+32	75	37	35	437	12,5	9,3	4,8	13,3
Cross passage	Km 0+20 – km 0+40	20	9	11	11	1	2,7	1,3	1,8
Left tube	Km 1.3+97 – km 1.5+44	147	72	179	2400	11,4	34,6	17,1	29,3
Total		242	118	225	2488	11,7			
